# The Effects of Exergaming on Sensory Reweighting and Mediolateral Stability of Women Aged Over 60: Usability Study

**DOI:** 10.2196/27884

**Published:** 2021-07-21

**Authors:** Mariann Sápi, Anna Fehér-Kiss, Krisztina Csernák, Andrea Domján, Sándor Pintér

**Affiliations:** 1 Doctoral School of Clinical Medicine University of Szeged Szeged Hungary; 2 Physiotherapy Center Albert Szent-Györgyi Health Center University of Szeged Szeged Hungary; 3 Department of Psychiatry and Psychiatric Clinic Bács-Kiskun County Hospital Kecskemét Kecskemét Hungary; 4 Department of Physiotherapy Faculty of Health Sciences and Social Studies University of Szeged Szeged Hungary

**Keywords:** exergaming, sensory reweighting, older women, mediolateral sway, vestibular

## Abstract

**Background:**

Older adults tend to experience difficulties in switching quickly between various reliable sensory inputs, which ultimately may contribute to an increased risk of falls and injuries. Sideward falls are the most frequent cause of hip fractures among older adults. Recently, exergame programs have been confirmed as beneficial tools for enhancing postural control, which can reduce the risk of falls. However, studies to explore more precisely which mechanism of exergaming directly influences older women’s ability to balance are still needed.

**Objective:**

Our aim was to evaluate, in a single-group pretest/posttest/follow-up usability study, whether Kinect exergame balance training might have a beneficial impact on the sensory reweighting in women aged over 60.

**Methods:**

A total of 14 healthy women (mean age 69.57 [SD 4.66] years, mean body mass index 26.21 [SD 2.6] kg/m^2^) participated in the study. The volunteers trained with the commercially available games of Kinect for Xbox 360 console 3 times (30 minutes/session) a week over a 6-week period (total of 18 visits). Participants’ postural sway in both the anteroposterior (AP) and mediolateral (ML) directions was recorded with NeuroCom Balance Master 6.0. To assess and measure postural sensory reweighting, the Modified Clinical Test of Sensory Interaction in Balance was used, where volunteers were exposed to various changes in visual (eyes open or eyes closed) and surface conditions (firm or foam surface).

**Results:**

In the ML direction, the Kinect exergame training caused a significant decrease in the sway path on the firm surface with the eyes open (*P*<.001) and eyes closed (*P*=.001), and on the foam surface with the eyes open (*P*=.001) and eyes closed (*P*<.001) conditions compared with baseline data. The follow-up measurements when compared with the baseline data showed a significant change in the sway path on the firm surface with the eyes open (*P*<.001) and eyes closed (*P*<.001) conditions, as well as on the foam surface with the eyes open (*P*=.003) and eyes closed (*P*<.001) conditions. Besides, on the firm surface, there were no significant differences in sway path values in the AP direction between the baseline and the posttraining measurements (eyes open: *P*=.49; eyes closed: *P*=.18). Likewise, on the foam surface, there were no significant differences in sway path values in the AP direction under both eyes open (*P*=.24) and eyes closed (*P*=.84) conditions.

**Conclusions:**

The improved posturography measurements of the sway path in the ML direction might suggest that the Kinect exergame balance training may have effects on sensory reweighting, and thus on the balance of women aged over 60. Based on these results, Kinect exergaming may provide a safe and potentially useful tool for improving postural stability in the crucial ML direction, and thus it may help reduce the risk of falling.

## Introduction

Slipping, tumbling, or any other kind of an unintentional loss of balance, which results in a fall and subsequent hospitalization due to injury, is a serious global concern for people over the age of 60 according to the World Health Organization [1].

It has been shown that age-related deficits can manifest in cognitive function [2], in neuromuscular control mechanisms [3,4], and in the following 3 sensory systems: the visual [5], the somatosensory [6], and the vestibular [7]. Various studies have shown that older individuals have a tendency to use proprioception rather than visual and vestibular cues for postural motor control. This dependence on the proprioceptive system also increases with age [3,8]. In direct contrast to this, Haibach et al [9] found that older adults tend to rely more heavily upon their visual input rather than the other sensory systems to compensate for age-related deficiencies.

According to previous studies [10,11], adults tend to experience difficulties in switching quickly between various reliable sensory inputs, which ultimately may contribute to an increased risk of falls. However, Allison et al [12] suggest that this particular process is not impaired among the target population as a direct result of aging. Regardless of whether sensory reweighting deteriorates or remains unchanged with age, therapists should aim to plan programs that can develop these previously mentioned sensory systems and thus decrease the risk of falls.

It has been confirmed that community-dwelling women over the age of 65 are at least two times as likely to suffer hip fractures due to a fall when compared with men [13]. In one study [14], osteoporosis-related fractures in Hungary were investigated and offered incidence data not only on hip, but also on several fractures between 1999 and 2003, when the total population was approximately 10 million inhabitants. According to the data reported in this 5-year period, 404,380 Hungarian women and 206,009 men over the age of 50 had at least one fracture. A possible reason behind this phenomenon might be attributed to the difference between each gender’s change in the level of sex hormones during various stages in life. The changes may contribute to older women having a more significant decrease in bone mineral density [15]. Besides age-related hormonal changes, multitasking increases women’s gait variability, and this has a direct relationship to the prevalence of falls [16]. Furthermore, elderly women with an abnormal balance while walking are more likely to fall [17]. According to the findings of Qazi et al [18], a static posturography test demonstrated that the mediolateral (ML) component of postural sway is most strongly associated with long-term fracture risk in postmenopausal women. In addition to that, sideward falls are the most frequent cause of hip fractures among older adults [19], meaning that it is of key importance to detect with posturography the quantifiable information on body sway that cannot be visible to the clinicians’ naked eyes [20]. Signs of instability are sometimes not immediately apparent in the clinical setting, but sensitive measurements, such as postural sway, can predict the likelihood of falls [18]. For this reason, it is essential to implement training programs that improve sensorimotor control in the critical ML direction.

Recently, exercise games that are played in a virtual, but realistic environment (exergames) have become popular in various fields of research. The use of different types of virtual reality (VR) systems has been considered a beneficial method to improve health gains in different populations and pathological conditions [21]. According to current systematic reviews, video game–based trainings help support physical health [22-25] and cognitive functions among older adults [26-28]. In the last decade nonimmersive VR (without the use of a head-mounted device) exergame trainings with the Kinect system have been proven to be favorable in improving postural control among older adults [29-33]. A recent study revealed significant effects on balance in older adults who had VR exercise training versus an inactive control group [34], as well as a conventional exercise training group [35]. However, the exact mechanism of action of exergaming in improving the balance ability of older adults is a complex process that remains unclear [26]. Thus, in order to provide sound recommendations for their clinical use, the authors suggested conducting further studies to explore more precisely which mechanism of exergaming directly influences an older individual’s ability to balance (in other words, what are the causes of the observed changes or what are the improvements from exergame interventions).

It has been suggested that one of the underlying effects of exergames might originate from sensory reweighting. Body sway–based assessments such as the Sensory Organization Test or the Modified Clinical Test of Sensory Interaction in Balance (m-CTSIB) are sensitive tools for measuring sensory feedback reactions and processes during static stance. These measurements can confirm changes in sensory reweighting following exergaming in patients with Parkinson disease [36], in healthy and young adults [37,38], in older adults [39], in healthy women [40], and in women with fibromyalgia [41].

In the past 3 years, the effect of exergaming on sensory reweighting among older women has received little attention despite its clinical importance for physiotherapists. Because of the limited number of studies available on this topic, this usability study is focused on examining the potential effects of a Kinect exergame training on sensory reweighting and balance in the ML direction in healthy older women.

## Methods

### Participants

For the purpose of this study, healthy, community-dwelling older women above the age of 60 were recruited via local announcements in the senior centers within the city of Szeged, Hungary. Exclusion criteria included self-reported comorbidities (such as cognitive impairment; disorders of the heart; circulatory, musculoskeletal, and respiratory ailments; autoimmune diseases; and neurological conditions), hearing or vision loss, prosthetics or artificial limbs, wounds or corns on lower extremities, and the use of medication that could affect balance or participation in other organized physical training exercise programs. Twenty active, community-based volunteers signed up for the training program; however, due to the exclusion criteria, only 14 of them could participate in the study. This study was performed according to the Declaration of Helsinki and was approved by the Ethics Committee of the University of Szeged, Hungary (registration No. 125/2015 SZTE). All participants gave their signed informed consent before participating in the training program.

### Training Protocol

The applied equipment consists of a motion-sensing RGB camera named Kinect (v1), an Xbox 360 console, and video games developed by Microsoft. During the training, pictures of the game’s scene and a player’s avatar were projected onto the wall via the camera’s full-body 3D motion capture.

Before the training program commenced, volunteers had not had any experience with exergames or any of the previously mentioned devices, and so it was important to have an introductory meeting prior to the first training session where instructions were given on how to play the gesture-controlled video games and an opportunity to experience them first-hand. The training took place at Albert Szent-Györgyi Clinical Center’s Physiotherapy Department 3 times a week over a 6-week period (total of 18 visits). These sessions were assisted by physiotherapists. Participants were instructed to wear a comfortable outfit and safe footwear for the 30-minute training. Games were chosen based on the type of movements their performance required, with the main aspect being that games had to contain patterns of everyday functional movements which modeled usual, frequent natural motions. Commercially available Kinect games were played by the participants which demanded continual displacement of the participants’ center of gravity (COG), transference of weight between lower limbs, and lateral trunk bending and frequent sidesteps. The motor stimulation during gameplay required balanced reactions and continuous postural adjustments associated with fast movement of the legs and arms. During the first half of the training sessions, games that consisted of more foreseeable movements and simple elements (eg, football, skiing) were played. Other more complex games that needed higher cognitive attention and fast reaction (20.000 Leaks, Space Pop, Reflex Ridge, River Rush) were selected to be played in the second half of the training sessions. All participants played the same type of games in pairs, in the same order on every training occasion, but were never allowed to play the same game on 2 consecutive training sessions. During the training, the players’ adaptation and progression, as well as the level of difficulty of the game, were continuously recorded and modified based on each participant’s overall ranking in the game. Between each game, there was approximately a 1-minute transition time so that players could take a short break.

### Measurement

In general, in order to assess an individual’s ability to both integrate various senses of balance and compensation, while 1 or more of these senses may be lacking [33], NeuroCom Balance Master 6.0 (Clackamas) and the m-CTSIB [42-44] were used. The posturography measurements were performed at 3 separate intervals: before the first training, after the completion of the training program (posttraining), and 6 weeks after the last training session (follow-up).

The Balance Master 6.0’s software provided the location of both the COG and center of pressure across all tests for the m-CTSIB. The m-CTSIB test was initially developed by Shumway-Cook and Horak [45] to differentiate sensory (somatosensory, visual, and vestibular) inputs involved in postural stability during a steady-state balance assessment, and it explored balance on various surface types, with and without vision, using 4 sensory conditions: (1) firm surface, eyes open; (2) firm surface, eyes closed; (3) foam surface, eyes open; and (4) foam surface, eyes closed. The results provided by the Balance Master 6.0’s software package gave 3 measurements of COG (3 × 10 s) in the anteroposterior (AP) and ML directions [35]. Based on a previous study [43] with elderly females in all 4 sensory conditions, this test had good to excellent reliability of ML (intraclass correlation coefficient 0.88-0.93) and AP path length (intraclass correlation coefficient 0.85-0.90).

For the assessment of balance on the foam surface, a NeuroCom square foam balance assessment pad (size 46 × 46 × 13 cm) was used. During the assessments, the base of support was fixed, and participants stood comfortably barefooted with arms to their side and their feet next to a mark on the platform. The measurements took place in a quiet room away from distractions.

### Data Analysis: Sway Path

The following equations were applied to calculate the sway paths in the ML and AP directions


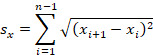



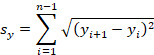


where *n* is the total number of samples; *i* is the sample number; *s_x_* is the path length of ML ways; and *s_y_* is the path length of the AP displacements of COG.

The following statistical analysis was conducted using Statistica 13 software (StatSoft). All sets of data were checked for normal distribution using the Kolmogorov–Smirnov test. Factorial analysis of variance was used to analyze sway data of the m-CTSIB test on firm and unstable (foam) surfaces to evaluate the main effects and the influences of the 2 visual conditions (eyes open and eyes closed) at all 3 time conditions (baseline, after the training, follow-up) as within-subjects factors. All values are given as mean (SD). The post hoc test was the Newman–Keuls test. A level of significance of *P*<.05 was applied.

## Results

Overall, 14 female volunteers (mean age 69.57 [SD 4.66] years, mean body mass index 26.21 [SD 2.6] kg/m^2^) participated in the study without any dropouts.

### Changes in Sway Path During Quiet Stance in the ML Direction

**Figure 1:**
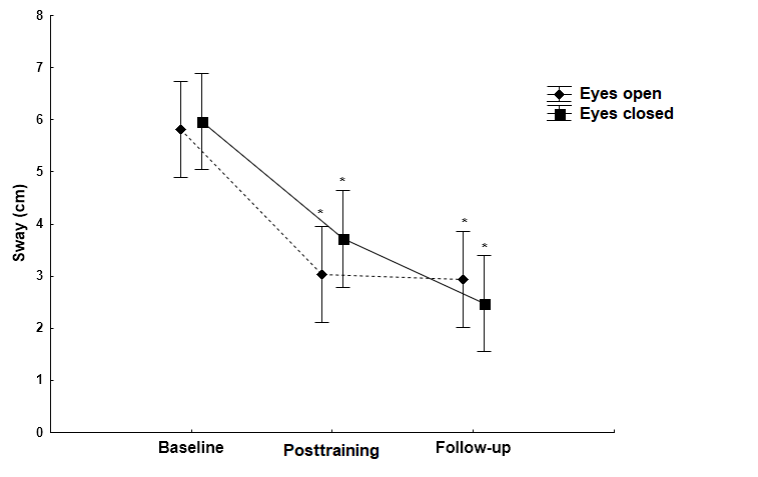
The effect of the Kinect training on sway path (mean [SD]) in the ML direction when standing on the firm surface with open and closed eyes. Statistically significant differences in sway path with eyes open (*P*<.001) and eyes closed (*P*=.001) posttraining conditions compared with the baseline data (asterisk). The follow-up measurements when compared with the baseline data showed statistically significant change in sway path on the firm surface with eyes open (*P*<.001) and eyes closed (*P*<.001) (asterisk). ML: mediolateral.

**Figure 2:**
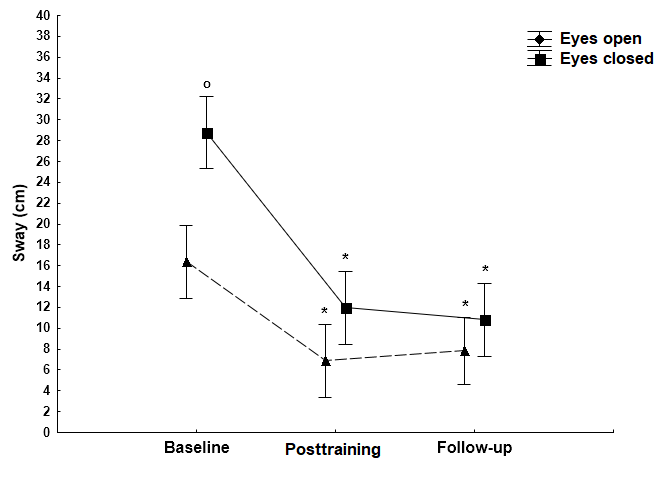
The effect of the Kinect training on sway path (mean [SD]) in the ML direction when standing on the foam surface with open and closed eyes. Statistically significant differences in sway path with eyes open (*P*=.001) and eyes closed (*P*<.001) posttraining conditions compared with the baseline data. The follow-up measurements when compared with the baseline data showed statistically significant changes in sway path with eyes open (*P*=.003) and eyes closed (*P*<.001) (asterisk). Statistically significant difference in sway path during baseline measurements with eyes closed (*P*<.001) compared with the eyes open condition (circle). ML: mediolateral.

In the ML direction, the Kinect exergame training caused a significant decrease in the sway path on the firm surface with eyes open (*P*<.001) and eyes closed (*P*=.001), and on the foam surface with eyes open (*P*=.001) and eyes closed (*P*<.001) conditions compared with the baseline data. The follow-up measurements when compared with the baseline data also showed significant change in the sway path on the firm surface with eyes open (*P*<.001) and eyes closed (*P*<.001), and on the foam surface with eyes open (*P*=.003) and eyes closed (*P*<.001; Figures 1 and 2). There were no significant differences in sway path values on the firm surface between eyes open and eyes closed conditions during the baseline (*P*=.81), after the training (*P*=.30), and follow-up (*P*=.48) evaluations. However, on the foam surface, results showed a significant interaction of vision × time for the sway path (*F*_2,246_=3.70, *P*=.02). Before the training, the sway path on the foam (unstable) surface with eyes closed was significantly longer (*P*<.001), whereas after the training the absence of visual information did not result in a significant increase (*P*=.16) of the sway path (Figure 2).

### Changes in Sway Path During Quiet Stance In the AP Direction

**Figure 3:**
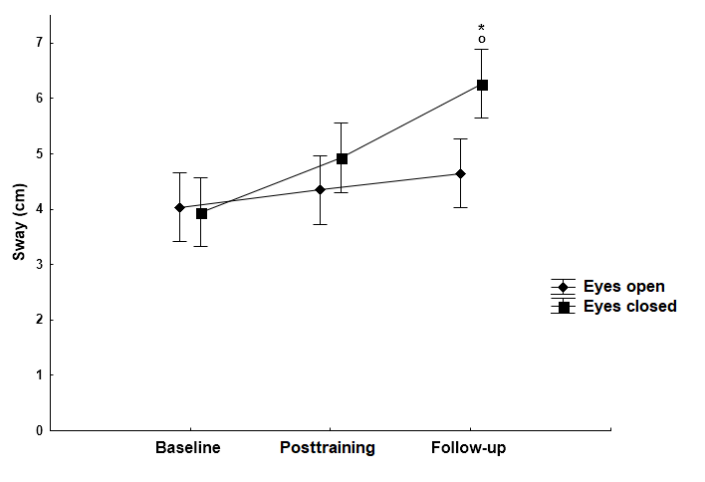
The effect of the Kinect training on sway path (mean [SD]) in the AP direction when standing on the firm surface with open and closed eyes. No statistically significant differences in sway path values on the firm surface between the baseline and posttraining measurements (eyes open [*P*=.49] and eyes closed [*P*=.18]). Statistically significant differences (*P*<.001) in comparison with the baseline measurement (asterisk) and in comparison with the open eye condition (circle) (*P*<.001). AP: anteroposterior.

**Figure 4:**
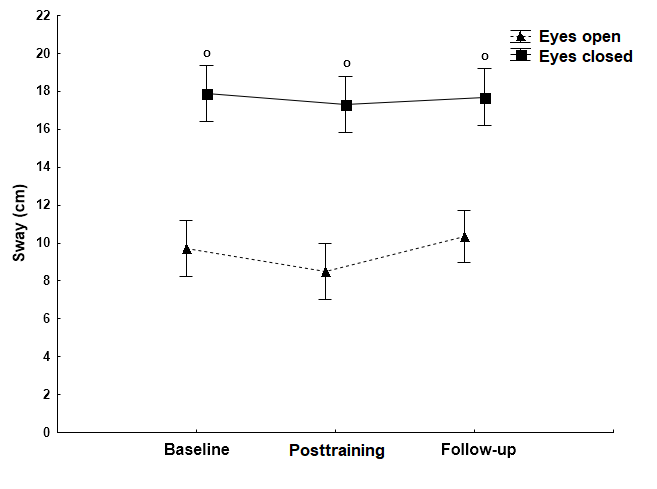
The effect of the Kinect training on sway path (mean [SD]) in the AP direction when standing on the foam surface with open and closed eyes. Statistically significant differences (*P*<.001) in comparison with the open eye condition (circle). AP: anteroposterior.

On the firm surface, there were no significant differences in sway path values in the AP direction between the baseline and the posttraining measurements (Figure 3; eyes open: *P*=.49; eyes closed: *P*=.18). Likewise, on the foam surface, there were no significant differences in sway path values in the AP direction under both eyes open (*P*=.24) and eyes closed (*P*=.84) conditions. During follow-up measurements, a main effect of vision was noted; in other words, closing the eyes resulted in a significant increase of the sway path (*P*<.001; Figure 3). On the unstable foam surface, a main effect of vision was observed and the absence of visual information significantly increased (*P*<.001) the sway path length in all time conditions (Figure 4).

## Discussion

### Principal Findings

Several studies have previously confirmed the beneficial effects of exergames on postural control among older adults [22,23,26,29,31-35]. This usability study shows that a simple Kinect game–based balance training might be beneficial for older women by improving balance in the ML direction. This study also demonstrates that exergaming might have a favorable effect in regards to the specific process of adjusting the sensory contributions to balance control [46], namely, sensory reweighting.

### Increased Lateral Stability

Based on our study results, an important finding is that the sway path in the ML direction on firm and foam surfaces, with eyes open and closed, improved statistically significantly, whereas no significant change was detected in the AP direction. However, decreased sway path indicates improved stability in the ML direction, which was concluded by Qazi et al [18] as the strongest component of postural sway predicting fractures in postmenopausal women. According to previous studies in the elderly population [47-49], ML sway can often be associated with risk of falls due to decreased proprioception and lower extremity muscle weakness in the lateral direction [50]. In light of the present findings following the training, improved sway results in the ML direction were observed when participants were standing on the foam surface with their eyes open. Significant decrease of ML sway might also implicate an improvement in proprioceptive function following the Kinect training. This finding is similar to the results of Sadeghi et al [51], which suggest that Kinect exergaming can improve proprioception by providing visual feedback and challenging motor skills and visual coordination.

### Improvement in Sensory Reweighting

An important finding of this paper is that the Kinect exergame training program significantly reduced postural sway on the foam surface with the eyes closed. Under this condition of the m-CTSIB, the central nervous system mostly relies on vestibular information [45]. In the review by Tahmosybayat et al [52] no exergame study has been presented that would train and assess sensory integration and sensory reweighting. Moreover, the authors suggested that the elements of sensory integration are too unsafe to be trained by disturbed sensory inputs during exergames. However, Roopchand-Martin et al [39] have examined the changes in m-CTSIB results following the Nintendo Wii Fit balance training in community-dwelling adults aged over 60. They found no significant results on the foam surface with the eyes closed condition after the training. By contrast, a Kinect-based physical exercise balance intervention in women with fibromyalgia has revealed significant improvements in the m-CTSIB with eyes closed on foam surfaces [41]. Another study [40] that examined the Wii Fit balance training for healthy women also found similar results: significant sensorimotor improvement in unilateral stance and limb strength. Nitz et al [41] concluded that these findings might not be surprising because the activities included in the Wii Fit training (such as yoga, balance, aerobic, and strength activities) involved considerable single-limb balance requirements and body weight–resistance movements. Although the aforementioned studies [40,41] investigated the effects of exergaming on balance in various sensory conditions with m-CTSIB, sensory reweighting following the trainings has not been proposed in these papers.

In contrast to these results, Yen et al [36] demonstrated that VR balance training significantly improved sensory reweighting in older adults with Parkinson disease when both visual and somatosensory inputs were unreliable. They have suggested that the VR training might be especially beneficial for fall prevention within this high-risk target group, as similar conditions may also occur in reality due to various extrinsic environmental risk factors (inappropriate footwear, poor lighting, slippery surfaces, loose rugs, or uneven steps) [36]. Other studies have found significant improvement in the eyes closed condition on unstable surface following a Wii Fit balance training among young adults [37] and healthy adults [38]. According to these studies [37,38], the reason for the improved vestibular function might be due to the quick displacement of COG in different directions, causing rapid changes in the head position. Similarly, during pretest measurements in this study, closing the eyes on the foam surface resulted in a statistically significant increase in the sway scores in the ML direction; however, posttraining measurements did not show deteriorated sway results. The reason for this might be that after the training, participants shifted to the remaining, accurate source of sensory information, and mainly relied on the vestibular system. Another possible explanation might be that during exergaming, participants had to complete several tasks containing movements such as reaching out and lateral steps while they needed to often change their head position.

Santos et al [53] have suggested that VR therapy enables patients to become immersed in an imaginary world, in which environmental perception is altered by artificial stimuli, thus resulting in a sensory conflict that can act on the vestibulo-ocular reflex (VOR). The central nervous system reacts to vestibular stimulus by reflexes such as the VOR, which stabilizes vision during head motion, and the vestibulospinal reflex, which induces a compensatory body motion to stabilize the head and body, and prevent falls [54]. Thus, types of exergames that require head movements in particular (rotation, lateral flexion, flexion) while players’ eyes are focusing (gazing) at one point can function as VOR training. Based on this study’s results, the applied Kinect games might improve sensory reweighting in favor of relying on vestibular inputs. In this study, while participants were playing the exergames, they had to keep their eyes on the screen while performing various head and limb movements.

### Lessons Learned From Kinect Exergames

Games such as 20, 000 Leaks, River Rush, Reflex Ridge, Super Saver Football mini-game, Space Pop, and Skiing might especially challenge the VOR because they require frequent head displacement movements. Additionally, these games could also improve stability in the lateral direction because of frequent weight shifting and sideward stepping. According to Swanenburg et al [55], exergaming that requires active stepping movements and that involves moving game projection is usable and facilitates gaze stability during head movements, which resulted in improved gait in healthy older adults. As balance is determined by various factors and maintained by complex processes, designing a balance training program requires precisely defining which target components or systems ought to be trained. Health care professionals might use exergames that could display participants’ changes of postural sway, reaction time, and limit of stability in various directions. Games which can train VOR by gaze stability during head movements should be provided for continuous monitoring to track players’ head movements. As falls occur mostly during activities of everyday life, exergames should be designed to involve functional movements that represent motions from daily life: alternately raising the feet from the ground (eg, stair stepping, stepping out of the bathtub), or reaching movements forward and sideways (eg, taking an item off a shelf below and above shoulder height, cleaning a window, hanging out the washing).

### Limitations

This usability study has encountered certain limitations as no sample size calculation was performed, and due to the lack of a control group and the relatively small sample size, the results should be interpreted cautiously. Therefore, these findings are not conclusive. Recruiting volunteers via local paper announcements for exergaming was not sufficient to get the attention we had hoped for. We believe that to attract more participants for future balance training programs, other types of advertisements should be used to generate interest. Posts on social media with video demonstrations and trials could raise interest especially among the youth, who could encourage their older relatives to participate. In this study, only older women participated, but to examine whether there is a gender difference in sensory reweighting following exergaming, future studies should also include a group of male participants. Investigating the effects of exergaming in older individuals with vestibular dysfunctions could also be beneficial, as this population is especially at risk of falling. Although there are studies that have described the positive effects of exergaming on balance ability [56,57] and on higher-order cognitive functions [58] when training independently at home, this study could not have been performed using a home-based exergame program. The reason for that is that the applied commercially available Kinect games are in English and no Hungarian translation is available. Therefore, participants needed assistance with starting and setting up the games, as well as technical help.

### Conclusions

In this usability study, women’s sway path decreased in the ML direction not only on the firm surface with eyes open, but also on the foam surface with eyes closed as a result of following the Kinect exergame training. These findings might support the idea that although the Kinect exergame training did not specifically contain direct challenging sensory tasks (eg, tilting or unstable surface or closed eyes exercises), the reduced sway results suggest that exergames could additionally result in sensory reweighting. The reason for this might be that the games contained tasks that needed constant gaze stabilizing and frequent head displacements. Therefore, this study’s improved sway results in the ML direction might contribute to decreased risk of falls among older women.

## Abbreviations

AP: anteroposterior
COG: center of gravity
m-CTSIB: Modified Clinical Test of Sensory Interaction in Balance
ML: mediolateral
VOR: vestibulo-ocular reflex
VR: virtual reality

## References

[1] World Health Organization (2021). Falls.

[2] Boisgontier MP, Beets IAM, Duysens J, Nieuwboer A, Krampe RT, Swinnen SP (2013). Age-related differences in attentional cost associated with postural dual tasks: increased recruitment of generic cognitive resources in older adults. Neurosci Biobehav Rev.

[3] Hurley M V, Rees J, Newham D J (1998). Quadriceps function, proprioceptive acuity and functional performance in healthy young, middle-aged and elderly subjects. Age Ageing.

[4] Nishihori Takezumi, Aoki M, Jiang Y, Nagasaki S, Furuta Y, Ito Y (2012). Effects of aging on lateral stability in quiet stance. Aging Clin Exp Res.

[5] Saftari LN, Kwon O (2018). Ageing vision and falls: a review. J Physiol Anthropol.

[6] Shaffer Scott W, Harrison A (2007). Aging of the somatosensory system: a translational perspective. Phys Ther.

[7] Anson E, Jeka J (2015). Perspectives on Aging Vestibular Function. Front Neurol.

[8] Wiesmeier IK, Dalin D, Maurer C (2015). Elderly Use Proprioception Rather than Visual and Vestibular Cues for Postural Motor Control. Front Aging Neurosci.

[9] Haibach PS, Slobounov SM, Newell KM (2008). The potential applications of a virtual moving environment for assessing falls in elderly adults. Gait Posture.

[10] Horak F, Shupert C, Mirka A (1989). Components of postural dyscontrol in the elderly: A review. Neurobiology of Aging.

[11] Teasdale N, Simoneau M (2001). Attentional demands for postural control: the effects of aging and sensory reintegration. Gait & Posture.

[12] Allison LK, Kiemel T, Jeka JJ (2006). Multisensory reweighting of vision and touch is intact in healthy and fall-prone older adults. Exp Brain Res.

[13] Finsterwald M, Sidelnikov E, Orav EJ, Dawson-Hughes B, Theiler R, Egli A, Platz A, Simmen HP, Meier C, Grob D, Beck S, Stähelin H B, Bischoff-Ferrari HA (2014). Gender-specific hip fracture risk in community-dwelling and institutionalized seniors age 65 years and older. Osteoporos Int.

[14] Péntek M, Horváth C, Boncz I, Falusi Z, Tóth E, Sebestyén A, Májer I, Brodszky V, Gulácsi L (2008). Epidemiology of osteoporosis related fractures in Hungary from the nationwide health insurance database, 1999-2003. Osteoporos Int.

[15] Fabrício Suzele Cristina Coelho, Rodrigues RAP, da Costa Moacyr Lobo (2004). [Falls among older adults seen at a São Paulo State public hospital: causes and consequences]. Rev Saude Publica.

[16] Johansson J, Nordström Anna, Nordström Peter (2016). Greater Fall Risk in Elderly Women Than in Men Is Associated With Increased Gait Variability During Multitasking. J Am Med Dir Assoc.

[17] Bergland A, Wyller T B (2004). Risk factors for serious fall related injury in elderly women living at home. Inj Prev.

[18] Qazi SL, Sirola J, Kröger Heikki, Honkanen R, Isanejad M, Airaksinen O, Rikkonen T (2019). High Postural Sway Is an Independent Risk Factor for Osteoporotic Fractures but Not for Mortality in Elderly Women. J Bone Miner Res.

[19] Fleps I, Vuille M, Melnyk A, Ferguson SJ, Guy P, Helgason B, Cripton PA (2018). A novel sideways fall simulator to study hip fractures ex vivo. PLoS One.

[20] Visser JE, Carpenter MG, van der Kooij H, Bloem BR (2008). The clinical utility of posturography. Clin Neurophysiol.

[21] Costa MTS, Vieira LP, Barbosa EDO, Mendes Oliveira L, Maillot P, Ottero Vaghetti César Augusto, Giovani Carta M, Machado S, Gatica-Rojas V, Monteiro-Junior RS (2019). Virtual Reality-Based Exercise with Exergames as Medicine in Different Contexts: A Short Review. Clin Pract Epidemiol Ment Health.

[22] Fang Q, Ghanouni P, Anderson SE, Touchett H, Shirley R, Fang F, Fang C (2020). Effects of Exergaming on Balance of Healthy Older Adults: A Systematic Review and Meta-analysis of Randomized Controlled Trials. Games Health J.

[23] Pacheco T, de Medeiros C S P, de Oliveira V H B, Vieira E R, de Cavalcanti F A C (2020). Effectiveness of exergames for improving mobility and balance in older adults: a systematic review and meta-analysis. Syst Rev.

[24] Zheng L, Li G, Wang X, Yin H, Jia Y, Leng M, Li H, Chen L (2020). Effect of exergames on physical outcomes in frail elderly: a systematic review. Aging Clin Exp Res.

[25] Xu W, Liang H, Baghaei N, Wu Berberich B, Yue Y (2020). Health Benefits of Digital Videogames for the Aging Population: A Systematic Review. Games Health J.

[26] Choi SD, Guo L, Kang D, Xiong S (2017). Exergame technology and interactive interventions for elderly fall prevention: A systematic literature review. Appl Ergon.

[27] Wollesen B, Wildbredt A, van Schooten Kimberley S, Lim Mei Ling, Delbaere Kim (2020). The effects of cognitive-motor training interventions on executive functions in older people: a systematic review and meta-analysis. Eur Rev Aging Phys Act.

[28] Zhao Y, Feng H, Wu X, Du Y, Yang X, Hu M, Ning H, Liao L, Chen H, Zhao Y (2020). Effectiveness of Exergaming in Improving Cognitive and Physical Function in People With Mild Cognitive Impairment or Dementia: Systematic Review. JMIR Serious Games.

[29] Bieryla KA (2016). Xbox Kinect training to improve clinical measures of balance in older adults: a pilot study. Aging Clin Exp Res.

[30] Silva KG, De Freitas TB, Doná Flávia, Ganança Fernando Freitas, Ferraz HB, Torriani-Pasin C, Pompeu JE (2017). Effects of virtual rehabilitation versus conventional physical therapy on postural control, gait, and cognition of patients with Parkinson's disease: study protocol for a randomized controlled feasibility trial. Pilot Feasibility Stud.

[31] Bacha Jéssica Maria Ribeiro, Gomes Gisele Cristine Vieira, de Freitas Tatiana Beline, Viveiro Larissa Alamino Pereira, da Silva Keyte Guedes, Bueno Géssika Costa, Varise Eliana Maria, Torriani-Pasin Camila, Alonso Angélica Castilho, Luna Natalia Mariana Silva, D'Andrea Greve Júlia Maria, Pompeu José Eduardo (2018). Effects of Kinect Adventures Games Versus Conventional Physical Therapy on Postural Control in Elderly People: A Randomized Controlled Trial. Games Health J.

[32] Kamińska Magdalena Sylwia, Miller Agnieszka, Rotter Iwona, Szylińska Aleksandra, Grochans Elżbieta (2018). The effectiveness of virtual reality training in reducing the risk of falls among elderly people. Clin Interv Aging.

[33] Ayed I, Ghazel A, Jaume-I-Capó Antoni, Moya-Alcover G, Varona J, Martínez-Bueso Pau (2018). Feasibility of Kinect-Based Games for Balance Rehabilitation: A Case Study. J Healthc Eng.

[34] Sadeghi H, Shojaedin S (2021). The Effect of virtual reality on postural stability and fall risk assessment of older women. Women?s Health Bulletin. Women's Health Bulletin.

[35] Sadeghi H, Jehu DA, Daneshjoo A, Shakoor E, Razeghi M, Amani A, Hakim MN, Yusof A (2021). Effects of 8 Weeks of Balance Training, Virtual Reality Training, and Combined Exercise on Lower Limb Muscle Strength, Balance, and Functional Mobility Among Older Men: A Randomized Controlled Trial. Sports Health.

[36] Yen C, Lin K, Hu M, Wu R, Lu T, Lin C (2011). Effects of virtual reality-augmented balance training on sensory organization and attentional demand for postural control in people with Parkinson disease: a randomized controlled trial. Phys Ther.

[37] Cone BL, Levy SS, Goble DJ (2015). Wii Fit exer-game training improves sensory weighting and dynamic balance in healthy young adults. Gait Posture.

[38] Cone BL, Goble DJ, Rhea CK (2017). Relationship between changes in vestibular sensory reweighting and postural control complexity. Exp Brain Res.

[39] Roopchand-Martin S, McLean R, Gordon C, Nelson G (2015). Balance Training with Wii Fit Plus for Community-Dwelling Persons 60 Years and Older. Games Health J.

[40] Collado-Mateo D, Gallego-Diaz JM, Adsuar JC, Domínguez-Muñoz F J, Olivares PR, Gusi N (2015). Fear of Falling in Women with Fibromyalgia and Its Relation with Number of Falls and Balance Performance. Biomed Res Int.

[41] Nitz JC, Kuys S, Isles R, Fu S (2010). Is the Wii Fit a new-generation tool for improving balance, health and well-being? A pilot study. Climacteric.

[42] Boughen J, Dunn K, Nitz J, Johnston V, Khan A (2013). A new method of interpreting the centre of gravity location using the modified Clinical Test of Sensory Interaction on Balance: A reliability study. Hong Kong Physiotherapy Journal.

[43] Rugelj D, Hrastnik A, Sevšek France, Vauhnik R (2015). Reliability of modified sensory interaction test as measured with force platform. Med Biol Eng Comput.

[44] Dawson N, Dzurino D, Karleskint M, Tucker J (2018). Examining the reliability, correlation, and validity of commonly used assessment tools to measure balance. Health Sci Rep.

[45] Shumway-Cook A, Horak F B (1986). Assessing the influence of sensory interaction of balance. Suggestion from the field. Phys Ther.

[46] Nashner L, Berthoz A (1978). Visual contribution to rapid motor responses during postural control. Brain Research.

[47] Maki BE, Holliday PJ, Topper AK (1994). A prospective study of postural balance and risk of falling in an ambulatory and independent elderly population. J Gerontol.

[48] Stel VS, Smit JH, Pluijm SM, Lips P (2003). Balance and mobility performance as treatable risk factors for recurrent falling in older persons. Journal of Clinical Epidemiology.

[49] Hilliard MJ, Martinez KM, Janssen I, Edwards B, Mille M, Zhang Y, Rogers MW (2008). Lateral balance factors predict future falls in community-living older adults. Arch Phys Med Rehabil.

[50] Lord S, Rogers M, Howland A, Fitzpatrick R (1999). Lateral stability, sensorimotor function and falls in older people. J Am Geriatr Soc.

[51] Sadeghi H, Hakim MN, Hamid TA, Amri SB, Razeghi M, Farazdaghi M, Shakoor E (2017). The effect of exergaming on knee proprioception in older men: A randomized controlled trial. Arch Gerontol Geriatr.

[52] Tahmosybayat R, Baker K, Godfrey A, Caplan N, Barry G (2018). Movements of older adults during exergaming interventions that are associated with the Systems Framework for Postural Control: A systematic review. Maturitas.

[53] Santos G, Zeigelboim DBS, Severiano M, Teive H, Liberalesso P, Marques J, Cordeiro M (2017). Feasibility of virtual reality-based balance rehabilitation in adults with spinocerebellar ataxia: a prospective observational study. Hearing, Balance and Communication.

[54] Fetter M (2007). Vestibulo-ocular reflex. Dev Ophthalmol.

[55] Swanenburg J, Wild K, Straumann D, de Bruin ED (2018). Exergaming in a Moving Virtual World to Train Vestibular Functions and Gait; a Proof-of-Concept-Study With Older Adults. Front Physiol.

[56] van Diest M, Stegenga J, Wörtche H J, Verkerke GJ, Postema K, Lamoth CJC (2016). Exergames for unsupervised balance training at home: A pilot study in healthy older adults. Gait Posture.

[57] Alhagbani A, Williams A (2021). Home-Based Exergames for Older Adults Balance and Falls Risk: A Systematic Review. Physical & Occupational Therapy In Geriatrics.

[58] Adcock M, Fankhauser M, Post J, Lutz K, Zizlsperger L, Luft AR, Guimarães Vânia, Schättin Alexandra, de Bruin ED (2019). Effects of an In-home Multicomponent Exergame Training on Physical Functions, Cognition, and Brain Volume of Older Adults: A Randomized Controlled Trial. Front Med (Lausanne).

